# Striking Oxygen Sensitivity of the Peptidylglycine α-Amidating Monooxygenase (PAM) in Neuroendocrine Cells*

**DOI:** 10.1074/jbc.M115.667246

**Published:** 2015-08-19

**Authors:** Peter D. Simpson, Betty A. Eipper, Maximiliano J. Katz, Lautaro Gandara, Pablo Wappner, Roman Fischer, Emma J. Hodson, Peter J. Ratcliffe, Norma Masson

**Affiliations:** From the ‡ Centre for Cellular and Molecular Physiology, University of Oxford, Oxford OX3 7BN, United Kingdom,; § Department of Neuroscience, University of Connecticut Health Center, Farmington, Connecticut 06030,; ¶ Fundacion Instituto Leloir, C1405BWE Buenos Aires, Argentina, and; ‖ Target Discovery Institute, University of Oxford, Oxford OX3 7FZ, United Kingdom

**Keywords:** copper monooxygenase, hypoxia, hypoxia-inducible factor (HIF), peptide hormone, secretion

## Abstract

Interactions between biological pathways and molecular oxygen require robust mechanisms for detecting and responding to changes in cellular oxygen availability, to support oxygen homeostasis. Peptidylglycine α-amidating monooxygenase (PAM) catalyzes a two-step reaction resulting in the C-terminal amidation of peptides, a process important for their stability and biological activity. Here we show that in human, mouse, and insect cells, peptide amidation is exquisitely sensitive to hypoxia. Different amidation events on chromogranin A, and on peptides processed from proopiomelanocortin, manifest similar striking sensitivity to hypoxia in a range of neuroendocrine cells, being progressively inhibited from mild (7% O_2_) to severe (1% O_2_) hypoxia. In developing *Drosophila melanogaster* larvae, FMRF amidation in thoracic ventral (Tv) neurons is strikingly suppressed by hypoxia. Our findings have thus defined a novel monooxygenase-based oxygen sensing mechanism that has the capacity to signal changes in oxygen availability to peptidergic pathways.

## Teaser

**Background:** Peptidylglycine α-Amidating Monooxygenase (PAM) is solely responsible for catalysis of amidation, a biologically important post-translational modification.

**Results:** Modification-specific antibodies reveal that peptide substrate amidation is strikingly sensitive to the exposure of cells to moderate hypoxia.

**Conclusion:** PAM-dependent amidation has the potential to signal oxygen levels in the same range as the hypoxia-inducible factor (HIF) system.

**Significance:** Physiological effects of hypoxia may be PAM-dependent.

## Introduction

In most organisms, oxygen is the ultimate electron acceptor in energy-generating biological oxidations. Maintenance of cellular oxygen homeostasis is therefore of fundamental importance, and involves systems that respond directly or indirectly (for instance, via the status of specific redox couples (1)), to the concentration of oxygen. Direct oxygen-sensing systems include those in which signals are generated by co-ordination of molecular oxygen (for instance to heme-proteins (2) and processes in which molecular oxygen is used as obligatory substrate in oxidation reactions that have a signaling function, as exemplified by the hypoxia inducible factor (HIF)^3^ system.

In mammalian cells, HIFs mediate a widely operative transcriptional response involving thousands of direct and indirect targets that adapt cells to hypoxic stresses (3). HIF is regulated by a series of 2-oxoglutarate dependent (2-OG) dioxygenases that catalyze the post-translational hydroxylation of specific prolyl and asparaginyl residues to promote both inactivation and proteasomal destruction of the transcriptional complex in the presence of oxygen (4). While protein hydroxylation by 2-OG dioxygenases has long been recognized in biosynthetic processes such as the stabilization of collagen (5), its use in signaling, as in the regulation of HIF was unprecedented. Given that the human genome encodes multiple types of oxygenase for which molecular oxygen is an obligatory co-substrate, these findings have generated interest in the possibility that one or more of these enzymes might also have the potential to signal oxygen levels in cells.

An important property of the HIF hydroxylase system is extreme sensitivity to hypoxia in cells (6, 7). Though the biophysical properties underpinning this sensitivity are not yet fully understood (8), the disposition of the enzyme/substrate couple in cells is such that HIF hydroxylation is clearly impaired under conditions of moderate cellular hypoxia. Although the HIF system is responsible for regulating many cellular processes, other responses to hypoxia, particularly those occurring very rapidly, appear to be independent of HIF-mediated transcription (9, 10). This has led to interest in defining other oxygen-sensitive pathways, which might be dependent on oxygenases whose activity on relevant substrates is also effectively restricted at moderate cellular oxygen concentrations.

To this end, we and others have studied the reactions of 2-OG dioxygenases that are closely similar to the HIF hydroxylases. While these studies have revealed new types of biological oxidation, to date these reactions appear substantially less sensitive to hypoxia than HIF hydroxylation. For instance, HIF is progressively induced in cells cultured in atmospheres ranging from 10% to 1% O_2_, whereas hydroxylation of ribosomal proteins by enzymes closely similar to the HIF hydroxylases is only suppressed by more severe hypoxia (<1% O_2_ (11, 12)). In an effort to define novel oxygen-sensitive pathways that have the potential to signal in a similar range to HIF, we have studied cellular responses to moderate hypoxia that appear to be distinct from those mediated by HIF.

In the course of this work we observed striking changes in the immunoactivity of chromogranin A (CgA), a precursor protein stored in the secretory granules of many neuroendocrine cells and have delineated a novel oxygen-sensitive signaling pathway. We show that C-terminal amidation of a range of peptides by the copper-dependent enzyme, PAM (peptidylglycine α-amidating monooxygenase) is strikingly sensitive to hypoxia in cells. Surprisingly, although PAM differs substantially from the HIF hydroxylases (13), its catalytic activity on a range of substrates, in different cell types, is at least as sensitive to hypoxia as HIF. Because PAM is essential during development (14, 15), and α-amidation is important for the bioactivity of peptide hormones and other molecules, our findings point to an important interface of these processes with cellular hypoxia.

## Experimental Procedures

### 

#### 

##### Cell Manipulations

Standard culture conditions were used for H727, H69, H146, Kelly, and AtT20 cells. All chemicals from Sigma except DMOG (Frontier Scientific), IOX2 and IOX3 (from Professor C. J. Schofield, Chemistry Dept., Oxford). Oligofectamine reagent (Invitrogen) was used for the transfection of 40 nm or 100 nm siRNA duplex twice at 24 h intervals. Cells were harvested and analyzed 24 h after the second transfection. Dharmacon On-TARGET plus SMARTpool reagents were used for CHGA (CgA), human and mouse PAM (and respective controls). HIF-1α and HIF-2α siRNA sequences are as previously described (16). Hypoxic incubations used an Invivo 400 workstation (Ruskinn Technology). For analysis of cell supernatants, cells were transferred to serum-free medium. For ascorbate supplementation, H727 cells were pre-incubated for 16 h in medium containing 1 mm ascorbate-2-phosphate (a long-acting ascorbate derivative). For copper supplementation, H727 cells were pre-incubated for 2 h in medium supplemented with 20 or 100 μm CuCl_2_ (17).

##### Immunoblotting

Cell extracts were prepared as follows: cells (4 × 10^6^) were washed in PBS and lysed in 300 μl of Igepal buffer (18) at 4 °C for 5 min. Samples were microfuged at 13,000 rpm for 5 min at 4 °C after which supernatant (cell extract) was retained in Laemmli sample buffer. Extracts were resolved by SDS-PAGE and electro-blotted onto PVDF membrane (Millipore). Primary antibodies used for analysis were: anti-human HIF-1α 1:1000 (clone 54, BD Biosciences) and anti-mouse HIF-1α 1:1000 (10009269, Cayman Laboratories); anti-γ-H2AX phospho S139 1:1000 (JBW301, Millipore); anti-β-actin HRP 1:10,000 (ab49900), anti-CgA C-term 1:5000 (EP1031Y), anti-CgA total 1:5000 (ab15160), and anti-human PAM 1:1000 EPR2643(2) all Abcam. Anti-PAM 1:1000 (JH629/630) (19) was affinity-purified. Antibody specific to the amidated C terminus of JP was generated using d-Tyr-Pro-Glu-Pro-Ser-Pro-Arg-Glu-NH_2_ (Anti-JP-NH_2_ 1:750 (#8, Jamie) (20), the addition of a Gly residue to the C terminus of this peptide (JP(12–19)) reduced its cross-reactivity 10,000-fold. An immunoglobulin-enriched fraction was incubated with JP(12–19) linked to Affi-Gel15 beads (Bio-Rad) to remove any antibodies capable of recognizing this peptide; the unbound fraction was affinity-purified using JP(12–18)NH_2_ linked to Affi-Gel10 beads. Preparation of anti-γ3-MSH 1:750 (JH189) (21, 22) was described previously; affinity purification was carried out using bovine γ3-MSH linked to Affi-Gel10 beads. For peptide analysis, 2 μg each peptide (dissolved in 20% DMSO) was dotted onto PVDF membrane prior to IB. CgA C-terminal blocking peptide for EP1031Y was from Abcam. For peptide-blocking experiments, peptides were used at 16 μg/ml in primary antibody solution. IB were visualized using ECL substrate and quantitated using either the Bio-Rad ChemiDoc MP Imaging system and Image Lab 5.0 software or densitometry analysis using NIH ImageJ.

##### MS/MS Analysis

CgA C-terminal blocking peptide was analyzed using an LC-MS/MS workflow consisting of nAcquity UPLC (Waters) and Orbitrap Velos mass spectrometer (Thermo Fisher). MS/MS spectra were searched using MASCOT (Matrix Science, v2.5).

##### RT-qPCR

Cells were lysed in TRIzol (Sigma), and mRNA extracted by phase separation. Equal amounts of mRNA template were used for cDNA synthesis using the High Capacity cDNA Kit (Applied Biosystems). Expression analyses were either performed using TaqMan® Assay (Applied Biosystems probes: CHGA Hs00900373.m1; CA9 Hs00154208.m1; LDHA Hs00855332.g1; SLC2A Hs00892681.m1) or SYBR Green on a StepOne thermocycler (both Applied Biosystems) using the ΔΔC_t_ method. Primers used for analysis of mouse PAM were (forward primer: CAGAACTATCCCAGAAGAGGC and reverse primer: TTCTGTTTCTTTGTGATGCCCA) and for HPRT (forward primer: AGCGTTTCTGAGCCATTGCT and reverse primer: GCTACCGCTCCGGAAAGC).

##### Immunofluorescence

*Drosophila* 1st instar larvae were collected and developed at 21, 5, 4, or 3% O_2_ in an Invivo_2_ 500 workstation (Ruskinn). Brains were then dissected at the 3rd instar and stained as reported previously (23). Briefly, larvae were dissected and fixed in 4% (*v*/*v*) formaldehyde for 20 min at room temperature, then incubated with primary antibody (rabbit anti-Pro FMRF (24) or rabbit anti-FMRF-NH_2_ (15) in PBT overnight at 4 °C. After washing and incubation with secondary antibody (goat anti-rabbit Cy3, Jackson #111-165-144) diluted 1:250 in PBT + 300 nm DAPI, brains were separated and mounted in 80% (*v*/*v*) glycerol in PBS for observation at the confocal microscope (Carl Zeiss LSM 510 Meta).

## Results and Discussion

**FIGURE 1.**
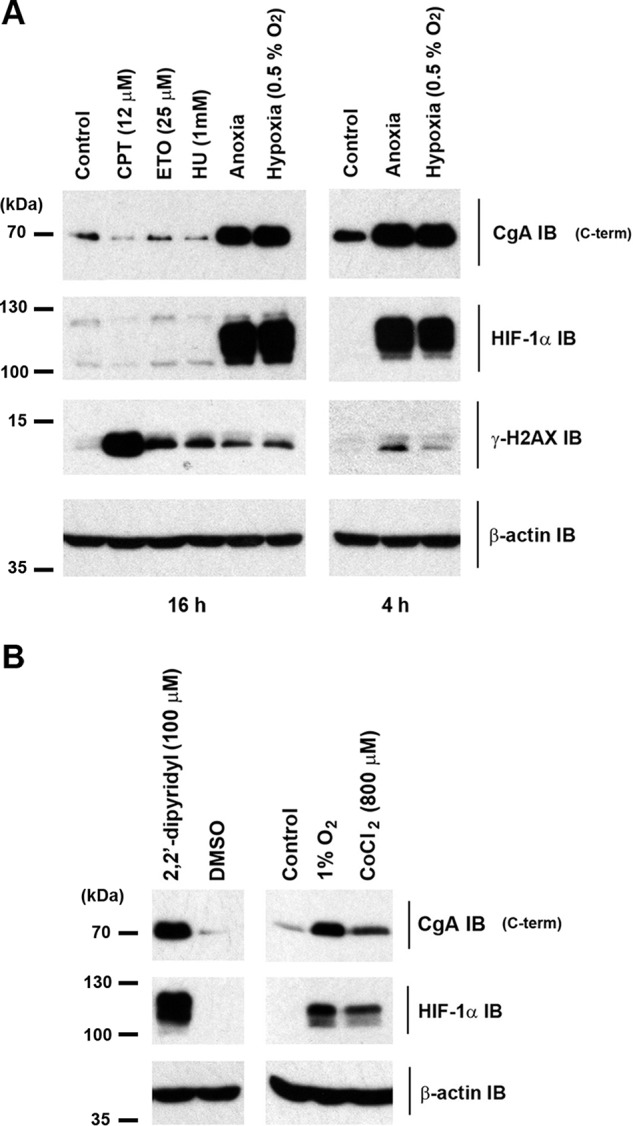
**Induction of CgA C-terminal immunoactivity by hypoxia, iron chelation, and cobalt in H727 cells.** *A*, cells were incubated under standard culture conditions (control), in hypoxia (0.5% O_2_), anoxia, or with agents that induce replication arrest *i.e.* camptothecin (*CPT*), etoposide (*ETO*), and hydroxyurea (*HU*) for the indicated times and extracts compared with control by IB. Efficacy of replication arrest was assessed by γ-H2AX phospho S139 IB. *B*, cells were incubated for 3 h with either 2,2′-dipyridyl or DMSO control (*left panel*) or in normoxia, 1% O_2_ or CoCl_2_ (*right panel*).

Our laboratory is interested in the regulation of biological processes by oxygen. In the course of analyzing oxygen-sensitive responses in the neuroendocrine cell line H727, we observed striking anoxia-dependent up-regulation of chromogranin A (CgA) immunoactivity (Fig. 1*A*). Other cellular stresses, such as agents that induce replication arrest, had no effect on this signal (Fig. 1*A*), whereas hypoxia (0.5% O_2_) induced CgA immunoactivity similarly. Treatment of cells with the iron-chelator, 2′2-dipyridyl, or with cobaltous ions, also induced CgA immunoactivity (Fig. 1*B*). These characteristics are similar to those established for HIF, which transduces oxygen-sensitive signals to diverse transcriptional targets; indeed, immunoblotting (IB) for the regulatory subunit of HIF, HIF-1α (Fig. 1, *A* and *B*) demonstrated an essentially identical pattern of inducible behavior, suggesting that CgA might be a target of the HIF pathway.

**FIGURE 2.**
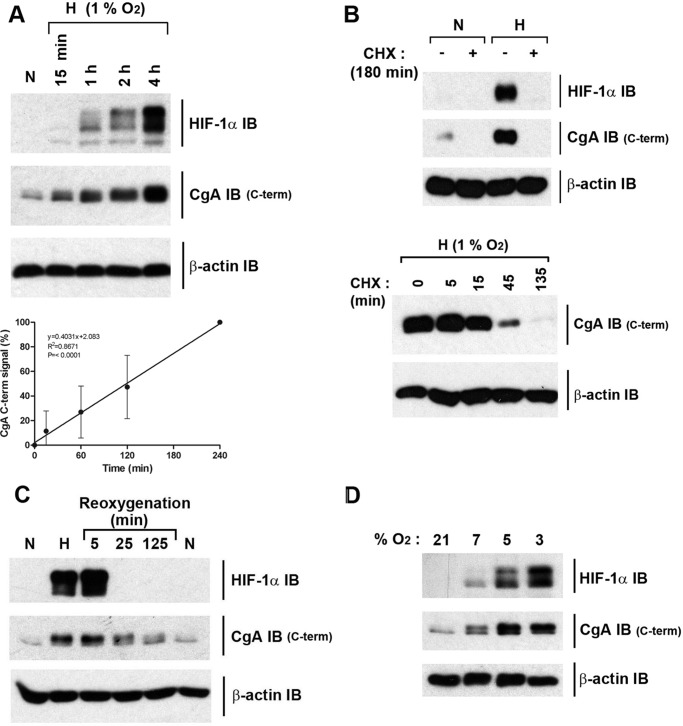
**Regulation of CgA C-terminal immunoactivity in H727 cells is rapid and highly responsive to changes in oxygen concentration.** *A*, *upper panel*, IB of extracts from cells in normoxia or at 1% O_2_ for indicated times. The CgA signal observed after a 15-min exposure of cells to 1% O_2_ is 11 ± 16% S.D. of the signal at 4 h. Quantitation based on *n* = 3 biological replicates. *Lower panel*, graph displaying the CgA IB data over the time course at 1% O_2_ (*n* = 3), indicating a linear response. *B*, protein synthesis is required for the CgA response to changes in oxygen concentration. *Upper panel*, IB of extracts from either control normoxic (*N*) or hypoxic cells (H, 1% O_2_ for 180 min) treated or not at *t* = 0 with 0.1 mm cycloheximide (*CHX*). Note that HIF-1α protein induction in hypoxia is prevented by cycloheximide treatment consistent with the known requirement for ongoing protein synthesis (39). *Lower panel*, hypoxic cells (3 h at 1% O_2_) were incubated under hypoxic conditions for a further 135 min prior to harvest with and without the addition of CHX (0.1 mm) for the indicated times. *C*, cells were incubated in normoxia, hypoxia (3 h at 1% O_2_), or hypoxia followed by re-oxygenation to 21% O_2_ for the indicated times. The HIF-1α IB signal is gone after 25 min reoxygenation. CgA C-terminal IB signal is also lost upon reoxygenation with 61 ± 4.6% S.D. of the *t* = 0 signal remaining at 125 min. Quantitation based on *n* = 3 biological replicates. *D*, IB of extracts from cells incubated for 3 h either in normoxia (21% O_2_) or graded hypoxia (7, 5, and 3% O_2_).

The characteristics of this oxygen-dependent regulation of CgA were then analyzed in more detail. Time-course studies indicated a rapid and linear response with induction of CgA (and HIF-1α) signal clearly detected by IB after only a 15 min exposure of cells to hypoxia (Fig. 2*A*). Thus, induction of CgA immunoactivity was at least as rapid or more rapid than that of HIF-1α (Fig. 2*A*, compare *upper* and *middle panels*). Treatment with cycloheximide blocked the hypoxic induction of CgA (Fig. 2*B*, *upper panel*) and when added after induction, led to a rapid loss of signal (Fig. 2*B*, *lower panel*). Re-oxygenation experiments also revealed a relatively rapid return to basal normoxic levels of CgA immunoactivity (Fig. 2*C*). Overall this indicates that the process generating the signal was dependent on new protein synthesis and was rapidly reversed in the presence of oxygen. The sensitivity across a range of different oxygen concentrations was also tested, and effects were observed even in moderate hypoxia (7% O_2_, Fig. 2*D*). In these experiments, parallel IB of CgA immunoactivity and HIF-1α indicated that the CgA response was at least as sensitive to oxygen as the HIF system.

**FIGURE 3.**
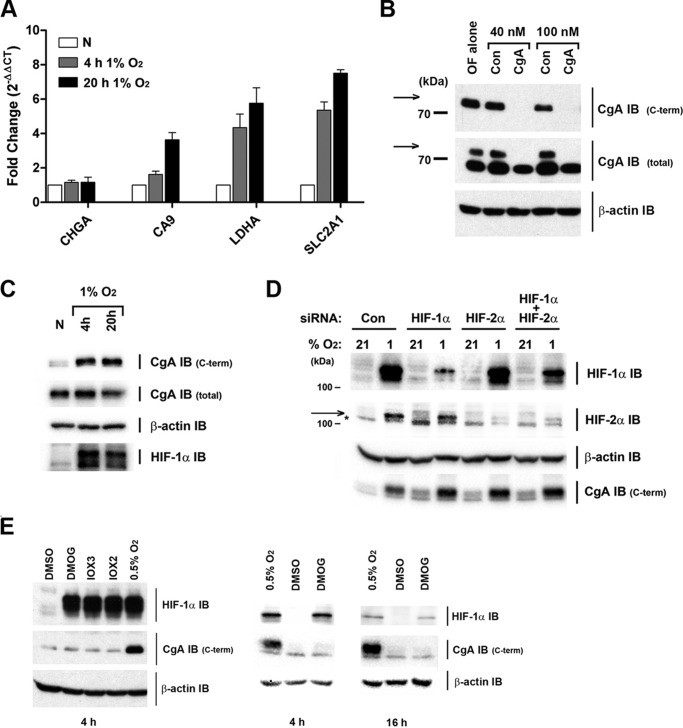
**Regulation of CgA by oxygen is post-translational and independent of the HIF system.** *A*, regulation of CgA by hypoxia is post-transcriptional. Control normoxic cells or cells incubated under hypoxia (1% O_2_) for either 4 or 20 h were harvested for mRNA analysis. RT-qPCR analysis shows chromogranin A (CHGA), CA9, LDHA, and GLUT1 (SLC2A1) mRNA levels normalized to cyclophilin and expressed as fold change (using the 2^−ΔΔCT^ method) relative to normoxic levels (*n* = 3 biological replicates). Data are mean ± S.D. *B*, verification of CgA antibody integrity. IB of normoxic extracts following treatment of cells with either oligofectamine alone (*OF*), or with siRNAs (either control or directed against human CgA). Signal with the CgA C-terminal antibody was specifically lost in extracts from CgA siRNA-treated cells. The CgA total antibody gave 2 bands (the upper of which co-migrated with that recognized by the CgA-C-terminal antibody and is marked with an *arrow*). Importantly, this upper immunoactive band was also not observed in extracts from CgA siRNA-treated cells. This confirmed that both CgA antibodies (C-terminal Ab and total Ab) could recognize CgA and assigned the upper band detected with total Ab, as CgA. *C*, IB of cells prepared in parallel with *A* for CgA C-terminal and total immunoactivity, β-actin and HIF-1α. *D*, IB of extracts from siRNA-treated cells exposed to 21 or 1% O_2_ for 20 h. The position of the HIF-2α immunoreactive band is marked with an *arrow* (* indicates a cross-reactive band). *E*, IB of cells incubated with vehicle control (DMSO), DMOG (1 mm), IOX3 (100 μm), IOX2 (125 μm), or at 0.5% O_2_ (with DMSO) for either 4 or 16 h.

The contribution of the HIF system to the regulation of CgA was therefore investigated directly. First, the effect of hypoxia on CgA mRNA was tested. RNA isolated from H727 cells incubated for 4 and 20 h in hypoxia (1% O_2_), was analyzed by RT-qPCR. Whereas the mRNA levels for three established HIF target genes (CA9, LDHA, and SLC2A1) increased during hypoxia, no change in CgA mRNA level was observed, thus arguing against a direct transcriptional (HIF-dependent) mechanism of regulation (Fig. 3*A*).

Other mechanisms of CgA regulation were then considered. As CgA undergoes extensive post-translational processing, the effects of hypoxia were re-examined using an independent CgA antibody. Whereas the first antibody (a monoclonal) was raised against a C-terminal peptide immunogen (residues 441–457), the second antibody was a polyclonal raised against a 194 amino acid immunogen located within residues 250–457 (CgA total, Fig. 3*B*). While a specific increase in CgA immunoactivity was again observed in hypoxia using this C-terminal antibody, the CgA signal determined using the CgA-total antibody was unaffected (Fig. 3*C*). Since CgA signals were of closely similar electrophoretic mobility, and the internal signal was unaffected by hypoxia, we hypothesized that one or more oxygen-dependent processing events were specifically affecting the epitope for the CgA C-terminal antibody. Quantitation by digital imaging of material from independent experiments (*n* = 4) revealed a 3.5-fold increase in CgA C terminus signal, relative to CgA internal (total) signal after 4 h of hypoxia.

In light of similar sensitivity to oxygen_,_ 2′2-dipyridyl, and cobaltous ions (Fig. 1*B*), we next examined the role of the HIF system in this post-translational regulation of CgA. The very rapid regulation of the CgA C-terminal immunoactivity by hypoxia (Fig. 2*A*) suggested that the mechanism was unlikely to be dependent upon HIF transcriptional activity and indeed after combined siRNA-mediated knockdown of HIF-1α and HIF-2α subunits, a robust hypoxic regulation of CgA C-terminal immunoactivity was still observed, confirming independence from HIF (Fig. 3*D*). An involvement of the oxygen-sensing HIF hydroxylases, all of which have been reported to act on non-HIFα substrates, was then tested using 2-OG-based inhibitors. Both the broad spectrum dioxygenase inhibitor dimethyloxalylglycine (DMOG) and more-specific prolyl hydroxylase inhibitors (IOX2 and 3, (25)) were used to treat normoxic cells. In all cases these inhibitors led to a robust induction of HIF-1α after 4 h, but had no effect on the CgA C-terminal signal (Fig. 3*E*, *left panel*). DMOG treatment for 16 h also had no effect on this CgA signal (Fig. 3*E*, *right panel*), thus excluding the possibility that effects may have been overlooked due to a differing kinetic action of the drugs, as compared with hypoxia. Taken together, these data indicate not only that oxygen-dependent post-translational regulation of CgA is HIF-independent, but also that it is independent of the known HIF hydroxylase oxygen-sensing enzymes, and probably independent of other 2-OG dioxygenases.

**FIGURE 4.**
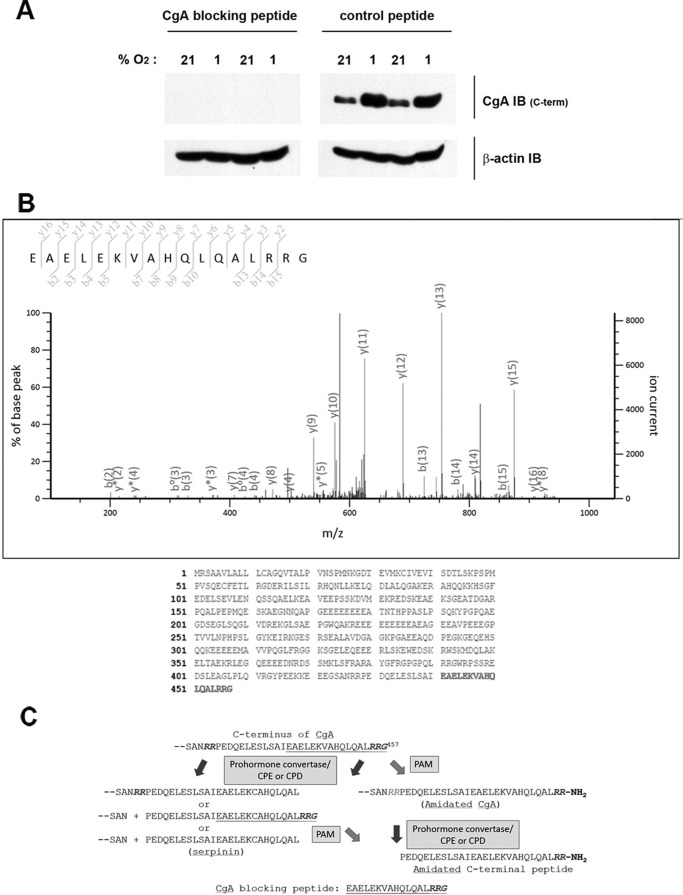
**Sequence assignment of the CgA C-terminal antibody peptide immunogen.** *A*, CgA C-terminal peptide specifically blocks signal from the CgA C-terminal antibody. Extracts from H727 cells incubated at 21% or 1% O_2_ for 4 h were IB with CgA C-terminal or β-actin antibody solution containing either control peptide or CgA C-terminal-blocking peptide. *B*, MS/MS assignment of the CgA C-terminal blocking peptide sequence. *Upper panel*, mascot annotated spectrum of the CgA C-terminal blocking peptide. *Lower panel*, amino acid sequence of CgA (Uniprot accession: P10645) with the C-terminal location of the peptide sequence highlighted in *bold. C*, schematic representation of possible post-translational processing events at the C terminus of CgA. Prohormone convertase cleavage (at dibasic amino acid recognition sites) followed by exopeptidase cleavage by enzymes such as carboxypeptidase E and D (CPE/CPD), can generate C-terminally truncated forms of CgA and the serpinin peptide. PAM-dependent amidation can generate amidated CgA or an amidated C-terminal peptide.

**FIGURE 5.**
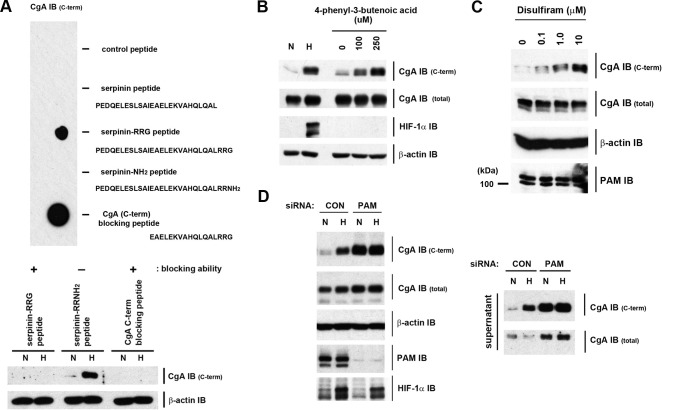
**Identification of PAM as an oxygen-sensor in H727 cells.** *A*, *upper panel*, indicated synthetic peptides spotted onto a membrane were IB using the CgA C-terminal antibody. *Bottom panel*, CgA C-terminal and β-actin antibodies were incubated with the indicated peptides during IB of cell extracts to test the ability of the peptides to block antigen recognition. Extracts were prepared from normoxic (*N*) cells or hypoxia cells (H, 1% O_2_ for 4 h). *B*, cells were incubated in normoxia, at 1% O_2_ or in the presence of 4P3BA for 4 h. *C*, cells were incubated with increasing doses of disulfiram. *D*, IB of extracts (*left panel*) and cell supernatants (*right panel*) prepared from control (*CON*) or PAM siRNA-treated cells incubated either in normoxia or at 1% O_2_ for 8 h.

Given the remarkably rapid and sensitive nature of this oxygen-dependent response we sought to identify the mechanism. The simplest explanation of the IB observations was that hypoxia may be inhibiting an oxygen-dependent modification of the C-terminal CgA epitope (hence recovering antibody recognition). Since this epitope was not known, it was necessary to define it experimentally. The blocking peptide provided by the manufacturer (Fig. 4*A*) was identified as the C-terminal 17 amino acids of CgA (Fig. 4*B*) a region of CgA that can undergo different types of post-translational processing (Fig. 4*C*). When tested directly in a dot blot format or indirectly by its ability to block the IB signal, cross-reactivity was confirmed (Fig. 5*A*). CgA is known to undergo extensive processing and the blocking peptide encompassed one of the sites for prohormone convertase mediated endoproteolytic cleavage (the dibasic amino acid site, -R-R-) and a site for C-terminal amidation (-R-G). As indicated in Fig. 4*C*, the amidation site can be eliminated by prohormone convertase cleavage. To further define the epitope, a panel of C-terminal peptides was generated. Cleavage by prohormone convertases followed by exopeptidases such as carboxypeptidase E or D (CPE or CPD), generates serpinin, a 26 amino acid peptide that lacks the C-terminal RRG sequence (26). When tested in the dot blot format (Fig. 5*A*, *upper panel*), synthetic serpinin failed to interact with the C-terminal CgA antibody. Thus the C-terminal -RRG sequence is an essential part of the epitope.

The α-amidation of secretory pathway proteins requires only a C-terminal -Gly (27) and α-amidation of the C terminus of CgA has been described (28, 29). This post-translational modification is catalyzed by PAM. PAM is a bifunctional enzyme; its copper-dependent peptidylglycine α-hydroxylating monooxygenase [PHM] domain converts peptidylglycine substrates to peptidyl-α-hydroxyglycine intermediates that are subsequently converted into amidated products plus glyoxylate by the zinc-dependent peptidyl-α-hydroxyglycine α-amidating lyase [PAL] domain (13). The reaction catalyzed by PHM results in the stereospecific incorporation of one atom of molecular oxygen into the substrate in a reaction that involves two single electron transfer steps (13). PAM-mediated C-terminal amidation occurs across a range of biologically active endocrine and nervous system peptides and in many cases has been shown to be required for normal biological activity *in vivo* (27). Given its requirement for molecular oxygen and its ability to modify the C-terminal residues of CgA, we tested amidation as the regulatory modification *in vitro*. Amidation of CgA would convert -RRG into -RRNH_2_ and the amidated synthetic C-terminal peptide (Serpinin-RRNH_2_) was not recognized by the CgA C-terminal antibody strongly suggesting that amidation was the oxygen-sensitive modification in cells (Fig. 5*A*).

**FIGURE 6.**
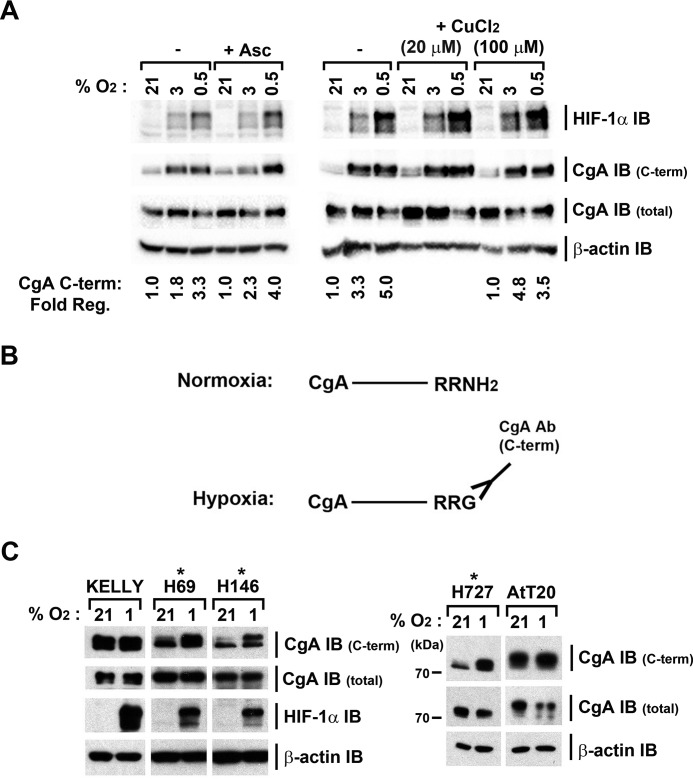
**PAM-dependent amidation of CgA is sensitive to oxygen in multiple cell lines and is not a consequence of cellular ascorbate or copper deficiency.** *A*, supplementation of culture medium with ascorbate or copper does not alter the sensitivity of PAM to oxygen concentration. H727 control, ascorbate- or copper-supplemented cells were exposed at the indicated oxygen concentrations for 4 h. Cell extracts were IB with the indicated antibodies and PAM activity assessed by changes in CgA C-terminal immunoactivity normalized to CgA total signal. Quantitation of the fold change (at 3% and 0.5% O_2_) relative to 21% O_2_ is shown for both control and ascorbate or copper supplemented samples. The addition of ascorbate did not alter the response of PAM to oxygen concentration, but did reduce the very low level of HIF-1α detected at 21% O_2_, in keeping with published observations (40). *B*, schematic representation of the PAM and oxygen-dependent amidation at the C terminus of CgA and its consequence for interaction with the CgA C-terminal antibody. In normoxia, amidation (or amidation followed by cleavage of C-terminal CgA residues) prevents recognition by the CgA C-terminal antibody. *C*, IB of extracts prepared from normoxic and hypoxic (4 h at 1% O_2_) cells indicates that oxygen-dependent processing of the C terminus of CgA occurs in multiple independent human lung neuroendocrine cell lines. Note that the CgA in the mouse AtT20 cell line has a higher molecular weight than that detected in H727 cells.

We therefore tested the effects of modulating PAM activity on the oxygen-sensitive CgA C-terminal signal. H727 cells were treated with varying doses of the PHM inhibitor, 4-phenyl 3-butenoic acid (4P3BA). 4P3BA is a substrate analogue and is reported to bind irreversibly to the active site of PAM. Treatment of cells with 4P3BA had no effect on CgA immunoactivity assayed with the internal antibody (Fig. 5*B*). However, the oxygen-responsive CgA C-terminal reactivity displayed clear dose-dependent responses both to 4P3BA and also to the PAM inhibitor and copper chelator, disulfiram (Fig. 5, *B* and *C*). These agents had no effect on the level of PAM protein. To test the role of PAM more specifically, we suppressed its expression using siRNA (Fig. 5*D*). IB with the CgA C-terminal antibody, revealed clear up-regulation of signal in normoxia (Fig. 5*D*) and ablation of the response to hypoxia, while IB with the internal antibody (CgA total) was unchanged. Similar results were obtained from both whole cell extracts and on secreted CgA detected in the culture supernatant (Fig. 5*D*, *lower panel*). To exclude the possibility that these results reflected PAM cofactor deficiencies in H727 cells that sensitized PAM to hypoxia, we re-tested this in the presence of ascorbate and copper supplements, but marked oxygen sensitivity was preserved (Fig. 6*A*). Our data thus identify PAM as a novel oxygen-sensor in this setting: PAM utilizes molecular oxygen to amidate the C terminus of CgA; in hypoxia, PAM cannot function, leading to the accumulation of intact, full-length CgA, which terminates with -RRG and can be recognized by the CgA C-terminal antibody (Fig. 6*B*).

To investigate the general function of PAM as an oxygen-sensor, we next analyzed other human and mouse neuroendocrine cell lines. IB analysis using the two CgA antibodies, identified additional human lung neuroendocrine cell lines (H69 and H146), that exhibited a specific increase in CgA C-terminal (non-amidated) signal in hypoxia (Fig. 6*C*). However this was not universal. In AtT20 and Kelly cells, CgA C-terminal immunoactivity was high in normoxic cells and was not increased by hypoxia (Fig. 6*C*), suggesting that these cells do not amidate full-length CgA.

**FIGURE 7.**
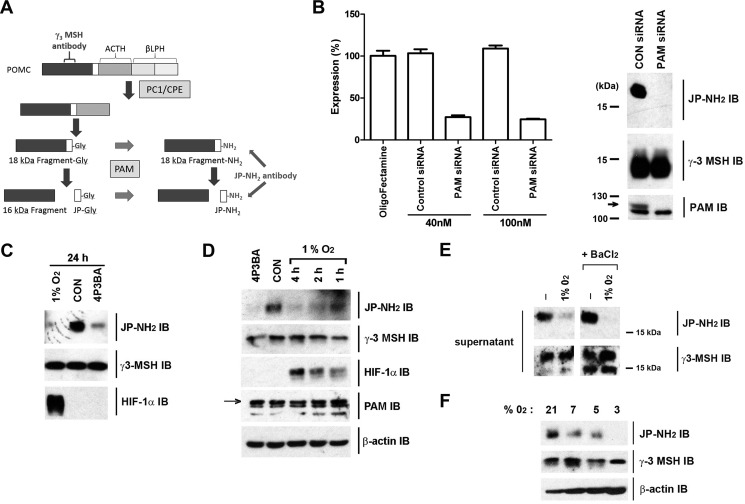
**PAM-dependent amidation of POMC peptides is sensitive to oxygen in AtT20 cells.** *A*, schematic representation of mJP processing from the N-terminal region of POMC illustrating the precursor fragments recognized by the γ3-MSH antibody (18-kDa Fragment-Gly, 18-kDa Fragment-NH_2_, and 16-kDa Fragment) and the JP-NH_2_ antibody (18-kDa Fragment-NH_2_ and JP-NH_2_). *B*, validation of PAM mRNA and protein knockdown following PAM siRNA treatment of AtT20 cells. RT-qPCR analysis (*left panel*) shows PAM mRNA levels normalized to HPRT and expressed as percentage expression relative to oligofectamine-treated cells (*n* = 3 technical replicates). Data are mean −/+ S.E. IB (*right panel*) of extracts prepared from control and PAM siRNA(40 nm)-treated AtT20 cells. The immunoactive band assigned as PAM by the siRNA analysis is marked with an *arrow*. The siRNA knockdown of PAM is accompanied by a loss of 18 kDa JP-NH_2_ immunoactivity with γ3-MSH immunoactivity unaffected. Note that the 16-kDa fragment (that is the predominant form detected by the γ3-MSH antibody in cell extracts) runs just below the 15-kDa marker on these gels. *C* and *D*, IB of AtT20 cell extracts either control (*CON*) or treated as indicated. 4P3BA was used at 250 μm. *E*, hypoxia inhibits amidation of constitutively secreted POMC 18-kDa fragment. IB of cell supernatants from normoxic or hypoxic AtT20 cells (1% O_2_ for 8 h) or parallel cells stimulated to secrete by incubation with BaCl_2_ (2 mm). The 18-kDa fragment-NH_2_ polypeptide is selectively removed from the regulated secretory pathway during secretory granule maturation and undergoes a constitutive-like secretion. In contrast, the 16-kDa fragment is retained with mJP in the mature granules and undergoes regulated secretion in response to secretagogues. These differential responses of the 18-kDa fragment and 16-kDa fragment were confirmed by IB using the γ3-MSH antibody while the JP-NH_2_ IB indicated the effect of hypoxia to reduce amidation status of the constitutively secreted 18-kDa Fragment-NH_2_. *F*, AtT20 cells were incubated for 4 h at the indicated oxygen concentrations.

Exactly why the C terminus of CgA is not amidated by PAM in all cell lines is unclear. For amidated full-length CgA to accumulate, PAM must be expressed and CgA cleavage into its many smaller products must proceed slowly. For the mouse pituitary corticotrope cell line AtT20, a lack of PAM activity can be excluded, as PAM is known to be responsible for amidation of its proopiomelanocortin (POMC)-derived products. AtT20 cells were therefore used to further explore the oxygen sensitivity of PAM. Mouse Joining Peptide (mJP), is the major POMC-derived amidated peptide produced in AtT20 cells. Both POMC and mJP can be detected in the same secretory granule (30). Analysis of mJP biosynthesis has demonstrated an ordered processing pathway involving an ∼18-kDa fragment derived from the N terminus of POMC, which has a C-terminal Gly and can be amidated by PAM (Fig. 7*A*, Ref. 22). This 18-kDa fragment is then cleaved to produce a ∼16-kDa fragment with release of mJP. Importantly the 18-kDa fragment is rapidly turned over (22) allowing effects on amidation to be studied directly (and by IB) without confounding signal from pre-existing stored polypeptide.

Using an antibody that is specific for the amidated form of mJP (JP-NH_2_), an 18-kDa fragment was indeed detected by IB of AtT20 cell extracts (Fig. 7*B*, *CON siRNA*). In parallel, cell extracts were immunoblotted using an antibody raised against an upstream region of POMC (γ3-MSH) as loading control. PAM-dependent changes in amidation were first confirmed by treatment of cells with PAM siRNA (Fig. 7*B*); while PAM siRNA had no effect on γ3-MSH signal, the 18-kDa JP-NH_2_ signal was completely ablated, confirming its identity (Fig. 7*B*). AtT20 cells were then incubated for 24 h either in the presence of the PAM inhibitor 4P3BA, or in 1% O_2_ (Fig. 7*C*). Both treatments ablated the 18-kDa JP-NH_2_ signal. Shorter time-course experiments demonstrated that hypoxia led to a relatively rapid inhibition of amidation, with loss of cellular 18-kDa JP-NH_2_ signal apparent after only 2 h in 1% O_2_ (Fig. 7*D*) and that secreted 18 kDa JP-NH_2_ signal was also affected (Fig. 7*E*). Importantly inhibition of 18-kDa fragment amidation was also seen in moderate hypoxia, including 7, 5, and 3% O_2_ (Fig. 7*F*). Thus PAM-dependent amidation manifested similar oxygen-sensitivity with two different substrates in two different cellular contexts.

**FIGURE 8.**
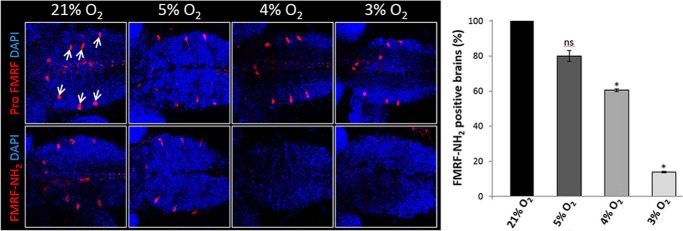
**Immunofluorescence of *Drosophila melanogaster* 3rd instar larval brains with antibodies to the FMRF pro-peptide (Pro FMRF) or to the amidated product, FMRF-NH_2_, merged with DAPI images.** *Drosophila* 1st instar larvae were developed under different oxygen concentrations and dissected at late 3rd instar for immunostaining. *Arrows* indicate Tv neurons, which are positive for FMRF (n > 30 brains in three independent experiments, Student's *t*-test. *, *p* < 0.001; *ns*: non-significant; bars represent S.E.). DAPI labels cell nuclei (*blue*).

The ability to respond to hypoxia is a fundamental physiological challenge faced by all species. Since the PAM system is conserved across invertebrate animal species (31) and the *peptidylglycine* α-*hydroxylating monooxygenase* (*PHM*) is essential for development in *Drosophila melanogaster* we sought to determine the oxygen-sensitivity of amidation *in vivo* in developing flies. To do so, we assessed the amidation status of FMRF neuropeptides that are specifically expressed in neuroendocrine Tv cells of larval brains (15). Brains from third instar larvae developed under different oxygen concentrations were analyzed by immunofluorescence using either FMRF-NH_2_ antibody or a control pro-FMRF antibody (Fig. 8). As expected, signal detected with these antibodies was specific to the Tv neurons. While the pro-FMRF antibody signal in the Tv neurons was not affected by hypoxia, the FMRF-NH_2_ signal was diminished at low oxygen concentrations, consistent with an inability of PHM to catalyze amidation under moderate hypoxia in this setting.

Overall our analyses of different PAM substrates, in different cell types and organisms reveal that PAM-dependent amidation in cells is very sensitive to changes in oxygen concentration and can operate within the same range as the cellular oxygen-sensing HIF hydroxylases. Because PAM-dependent amidation (like HIF hydroxylation) is irreversible, bi-directional responses that rapidly up-regulate and down-regulate levels of amidation can only be observed on rapidly turned-over PAM substrates. The best characterized PAM substrates are the (neuro)-endocrine peptides that are stored long-term within secretory granules. Although amidation of these substrates may be altered by sufficiently long-term hypoxia (*e.g.* Fig. 8), an acute oxygen-signaling function of PAM is unlikely to occur in this setting due to the confounding effects of peptide storage. In keeping with this, we have so far only observed modest effects of short-term hypoxia (24 h) on the amidation status of stored peptides assayed by mass spectrometry, but an increase in effect following the depletion of stored peptide reserves. Instead our data points to an involvement of amidation in acute oxygen-signaling processes through the regulation of PAM substrates that undergo constitutive (or constitutive-like) secretion and that are rapidly replenished (as we have observed for CgA, Fig. 2 and for the 18-kDa JP-NH_2_, Fig. 7). To date, most analyses of amidation have focused on the activity and stability of peptides after release from storage granules, and the function of amidation in constitutive secretory pathways has not been explored. With respect to CgA, circulating CgA (and fragments thereof) are reported to form a balance of pro- and anti-angiogenic factors (32) but any effect of amidation status in determining this balance is currently unclear. In relation to the potential for transducing oxygen-sensitive reactions, it is also of interest that PAM has other non-peptide (*e.g.* fatty acid) substrates (33, 34) and that PAM expression is not restricted to neuroendocrine cell types (35, 36). Importantly, phylogenetic analyses have also concluded that PAM predates the origins of the nervous and endocrine systems (31), again pointing to an important role for amidation in other conserved signaling pathways.

Overall, our study of rapidly turned-over PAM substrates has revealed the unexpected and remarkable sensitivity of cellular PAM to oxygen concentration and points to a potential role for PAM-dependent amidation in direct oxygen-sensing. Previous analyses have suggested additional interfaces with hypoxia *e.g.* an increase in PAM activity after chronic intermittent hypoxia was proposed to reflect differential proteolytic processing of PAM (37) and increases in PAM mRNA have also been observed after 24 h of hypoxia (38). However, our novel finding that amidation can be profoundly restricted by acute hypoxia may now inform on the other cellular roles of PAM that are already indicated, but poorly understood.

## Author Contributions

P. D. S. performed experiments, analyzed data and contributed to the preparation of figures and text. E. J. H. provided technical assistance. R. F. performed experiments shown in Fig. 4*B*. M. J. K. and L. G. performed and P. W. designed and analyzed the experiments shown in Fig. 8. B. A. E. provided reagents, analyzed data, and contributed to the preparation of figures and text. P. J. R. designed, analyzed, and wrote the manuscript. NM designed, performed, analyzed, prepared figures, and wrote the manuscript. All authors reviewed the results and approved the final version of the manuscript.

## Footnotes

3 The abbreviations used are:

HIF: hypoxia-inducible factor
2-OG: 2-oxoglutarate
4P3BA: 4-phenyl 3-butenoic acid
CgA: chromogranin A
DMOG: dimethyl oxalylglycine
mJP: mouse Joining Peptide
PAM: peptidylglycine α-amidating monooxygenase
PHM: peptidylglycine α-hydroxylating monooxygenase
POMC: proopiomelanocortin
Tv: thoracic ventral.
